# Underlying mechanisms of glucocorticoid-induced β-cell death and dysfunction: a new role for glycogen synthase kinase 3

**DOI:** 10.1038/s41419-021-04419-8

**Published:** 2021-12-07

**Authors:** Etienne Delangre, Junjun Liu, Stefania Tolu, Kamel Maouche, Mathieu Armanet, Pierre Cattan, Gaëlle Pommier, Danielle Bailbé, Jamileh Movassat

**Affiliations:** 1 Université de Paris, BFA, UMR 8251, CNRS, Team « Biologie et Pathologie du Pancréas Endocrine », Paris, France; 2 Cell Therapy Unit, Saint-Louis hospital, AP-HP, and Université de Paris, Paris, France; 3 Present Address: Shandong Institute of Endocrine & Metabolic Diseases, Shandong First Medical University & Shandong Academy of Medical Sciences, Jinan, China

**Keywords:** Cell biology, Cell death

## Abstract

Glucocorticoids (GCs) are widely prescribed for their anti-inflammatory and immunosuppressive properties as a treatment for a variety of diseases. The use of GCs is associated with important side effects, including diabetogenic effects. However, the underlying mechanisms of GC-mediated diabetogenic effects in β-cells are not well understood. In this study we investigated the role of glycogen synthase kinase 3 (GSK3) in the mediation of β-cell death and dysfunction induced by GCs. Using genetic and pharmacological approaches we showed that GSK3 is involved in GC-induced β-cell death and impaired insulin secretion. Further, we unraveled the underlying mechanisms of GC-GSK3 crosstalk. We showed that GSK3 is marginally implicated in the nuclear localization of GC receptor (GR) upon ligand binding. Furthermore, we showed that GSK3 regulates the expression of GR at mRNA and protein levels. Finally, we dissected the proper contribution of each GSK3 isoform and showed that GSK3β isoform is sufficient to mediate the pro-apoptotic effects of GCs in β-cells. Collectively, in this work we identified GSK3 as a viable target to mitigate GC deleterious effects in pancreatic β-cells.

## Introduction

Glucocorticoids (GCs) are steroid hormones produced by the adrenal gland, under the control of the hypothalamo-pituitary axis [1]. They regulate a large number of physiological processes and are widely used in therapy for their anti-inflammatory, immuno-modulatory and anti-allergic properties [2]. Their clinical usage remains irreplaceable, especially in case of transplantation [3], or for treating chronic inflammatory diseases such as rheumatoid arthritis [4], asthma [5] or multiple sclerosis [6]. GCs effects are mainly mediated by the glucocorticoid receptor (GR), which upon activation and nuclear translocation acts as a transcription factor, regulating the expression of a large spectrum of genes. In the absence of GCs, the GR is mainly located in the cytoplasm, in a macromolecular complex with chaperone proteins [7]. Its ligand-dependent nuclear translocation is known to be regulated by post-translational modifications, especially by phosphorylation [8].

Unfortunately, the therapeutic benefits of GCs are accompanied with serious side effects, particularly in the context of chronic treatments. Osteoporosis [9], loss of muscular mass [10] or a disruption in glucose metabolism, including diabetes [11] are among the most common side effects which are also found in Cushing’s disease suffering patients [12].

The exact incidence of GC-induced diabetes is unknown. Hjelmesaeth and coworkers have shown that about 20% of patients treated with GC-based therapy after renal transplantation develop diabetes [13]. Overall, up to 2% of the cases of diabetes might be associated with GC therapies [14]. Moreover, in human, one single dose of 75 mg prednisolone, a synthetic GC, induced glucose intolerance [15]. GCs are known to have adverse effects on pancreatic β-cell homeostasis, affecting their development [16], their secretory function [17, 18] and their survival [19]. These defects could contribute to the pathogenesis of GC-induced diabetes.

A better understanding of the complex regulation of GC signaling and its crosstalk with other intracellular pathways may provide the opportunity to minimize the undesirable effects of GCs and improve their benefit-risk profile for prolonged therapeutic applications.

In this study, we investigated the possible implication of Glycogen Synthase Kinase 3 (GSK3) in the mediation of diabetogenic effects of GCs in β-cells. GSK3 is a serine/threonine kinase, initially described as the kinase inhibiting Glycogen Synthase in the insulin signaling pathway [20]. GSK3, with two isoforms, GSK3α and GSK3β, encoded by two different genes, is implicated in a large variety of cellular processes, including proliferation, migration, and metabolism [21]. In some cellular systems, both in rodents [22] and humans [23], GSK3 has been reported to interact with the GC signaling pathway through the regulation of GR nuclear localization and the modulation of its transcriptional activity. However, to date, despite the independent roles of GCs and GSK3 in β-cell biology, the interplay between these two crucial pathways has not been elucidated in these cells. In this study, through pharmacological and genetic inhibition of GSK3, we investigated its role in the mediation of deleterious effects of GCs in β-cells. We showed for the first time that GSK3 is implicated in the adverse effects of GCs on survival and the secretory function of pancreatic islets. Moreover, we dissected some of the mechanisms underlying the crosstalk between GSK3 and GCs pathway, and uncovered the proper contribution of GSK3α and GSK3β isoforms to this process.

## Results

### GSK3 inhibition protects β-cells from Dexa-induced cell death

**Fig. 1:**
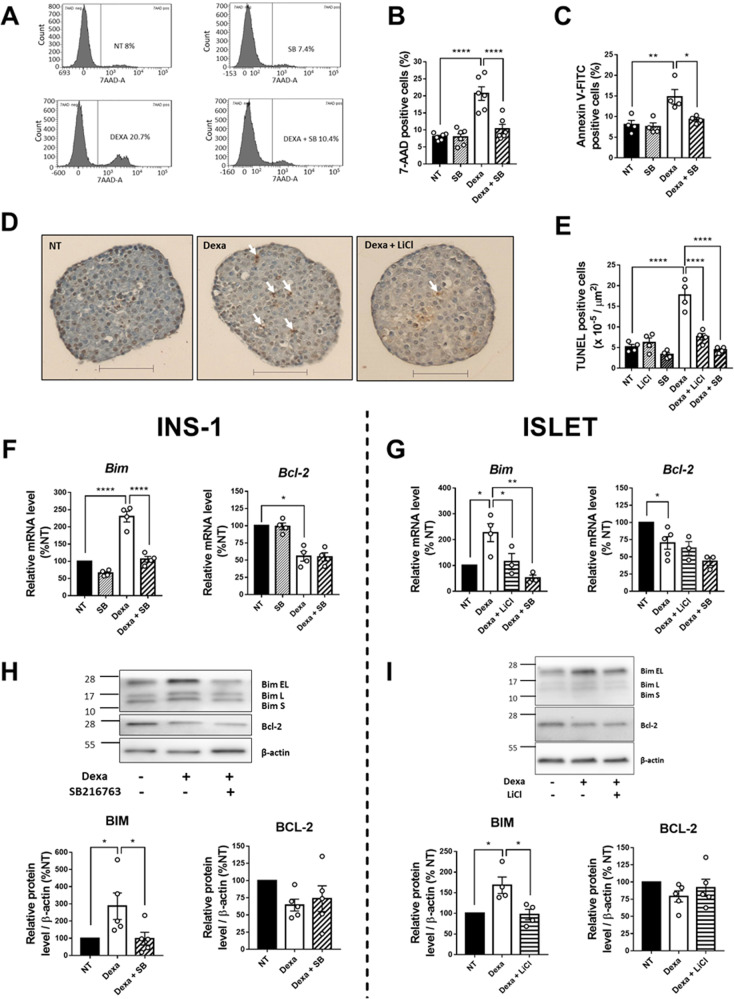
Treatment with LiCl or SB216763 decreases Dexa-induced cell death in INS-1 832/13 cell line and in rat islets. **A** Cell death was assessed by 7-AAD incorporation measured by flow cytometry after 24 h incubation with or without Dexamethasone (Dexa) and 27 h incubation with or without SB216763 (SB) in INS-1 832/13 cells. **B** Quantification of 7-AAD positives INS-1 832/13 cells (*n* = 6). **C** Cell apoptosis was evaluated by Annexin V-FITC incorporation assay. Annexin V-FITC positive cells were measured by flow cytometry after 24 h incubation with or without Dexamethasone (Dexa) and 27 h incubation with or without SB216763 (SB) in INS-1 832/13 cells (*n* = 4). **D** Representative images of TUNEL-positive cells in islets, after 24 h incubation with or without Dexamethasone (Dexa), and 27 h incubation with or without LiCl. The TUNEL-positive nuclei are stained in brown. White arrows show positive cells. Scale bar: 200 µm. **E** Quantification of TUNEL-positive cells in islets (*n* = 4) after 24 h incubation with or without Dexamethasone (Dexa) and 27 h incubation with or without LiCl or SB216763 (SB). More than 20 islets per condition were counted. **F** Relative mRNA levels of *Bim* and *Bcl-2*, measured by qPCR (*n* = 4) in INS-1 832/13, after 24 h incubation with or without Dexamethasone (Dexa) and 27 h incubation with or without SB216763 (SB). Results are expressed as percentage of NT condition for each independent experiment. **G** Relative mRNA levels of *Bim* and *Bcl-2*, measured by qPCR (*Bim*: *n* = 3–4; *Bcl-2* = 3–5) in islets, after 24 h incubation with or without Dexamethasone (Dexa) and 27 h incubation with or without LiCl or SB216763 (SB). Results are expressed as percentage of NT condition for each independent experiment. Cyclophilin A is used as housekeeping gene in figure **F** and **G**. **H** Representative Western Blot (top) and quantification (bottom) of Bim and Bcl-2 protein levels in INS-1 832/13 cells after 24 h incubation with or without Dexamethasone (Dexa) and 27 h incubation with or without SB216763 (SB) (*n* = 5). β-actin is used as loading control. Results are expressed as percentage of NT condition for each independent experiment. **I** Representative Western Blot (top) and quantification (bottom) of Bim and Bcl-2 protein levels in islets after 24 h incubation with or without Dexamethasone (Dexa) and 27 h incubation with or without LiCl (Bim *n* = 4; Bcl-2 *n* = 5). β-actin is used as loading control. Results are expressed as percentage of NT condition for each independent experiment. Results are shown as means ± S.E.M. *n* represents the number of independent experiments. *p < 0.05; **p < 0.01; ****p < 0.0001.

First, we examined the effect of dexamethasone (Dexa), a widely used GC analog, on cell apoptosis in the β-cell line INS-1 832/13. After 24 h of treatment, Dexa induced cell death, as reflected by the increased incorporation of 7-AAD in INS-1 832/13 cells (Fig. 1A, B). Interestingly, the inhibition of GSK3 by SB216763 reduced the Dexa-induced cell death, while it had no effect on cell death in cells not exposed to Dexa. This effect on cell apoptosis was confirmed by the Annexin V-FITC incorporation assay (Fig. 1C and Supplemental Fig. S1).

Next, in order to investigate cell apoptosis induced by Dexa in pancreatic islets isolated from Wistar rats, we performed TUNEL staining in islet sections (Fig. 1D, E). Dexa strongly increased the number of TUNEL positive cells in islets. The treatment of islets with GSK3 inhibitors, either LiCl or SB216763, significantly decreased the Dexa-induced apoptosis in islet cells, as reflected by a significant decrease of the number of TUNEL-positive cells, reaching a level similar to that observed in control islets which were not treated with Dexa. These results suggest the implication of GSK3 in the induction of cell death by Dexa in β-cells.

Apoptosis is regulated by the Bcl-2 family of proteins, including pro-apoptotic factors such as Bim or anti-apoptotic factors such as Bcl-2. Here, in INS-1 832/13 and in islets, Dexa increased mRNA and protein levels of Bim and decreased those of Bcl-2, suggesting the activation of apoptosis (Fig. 1F–I). The inhibition of GSK3 in INS-1 832/13 and islets, either with LiCl or with SB216763 abrogated the over-expression of Bim induced by Dexa, whereas it did not change the down-regulation of Bcl-2.

It has been reported that the GC-induced apoptosis is associated with the increase of oxidative stress in β-cells [24]. We assessed the expression of some of the mediators of these pathways in INS-1 832/13 cells. We found no significant changes in the expression of *NOX4*, *SOD1*, *SOD2*, in cells treated with Dexa. Moreover, the expression of *CHOP*, a marker of ER stress was unaffected following Dexa treatment (Supplemental Fig. S2).

### GSK3 inhibition prevents the Dexa-induced impairment of insulin secretion in islets

**Fig. 2:**
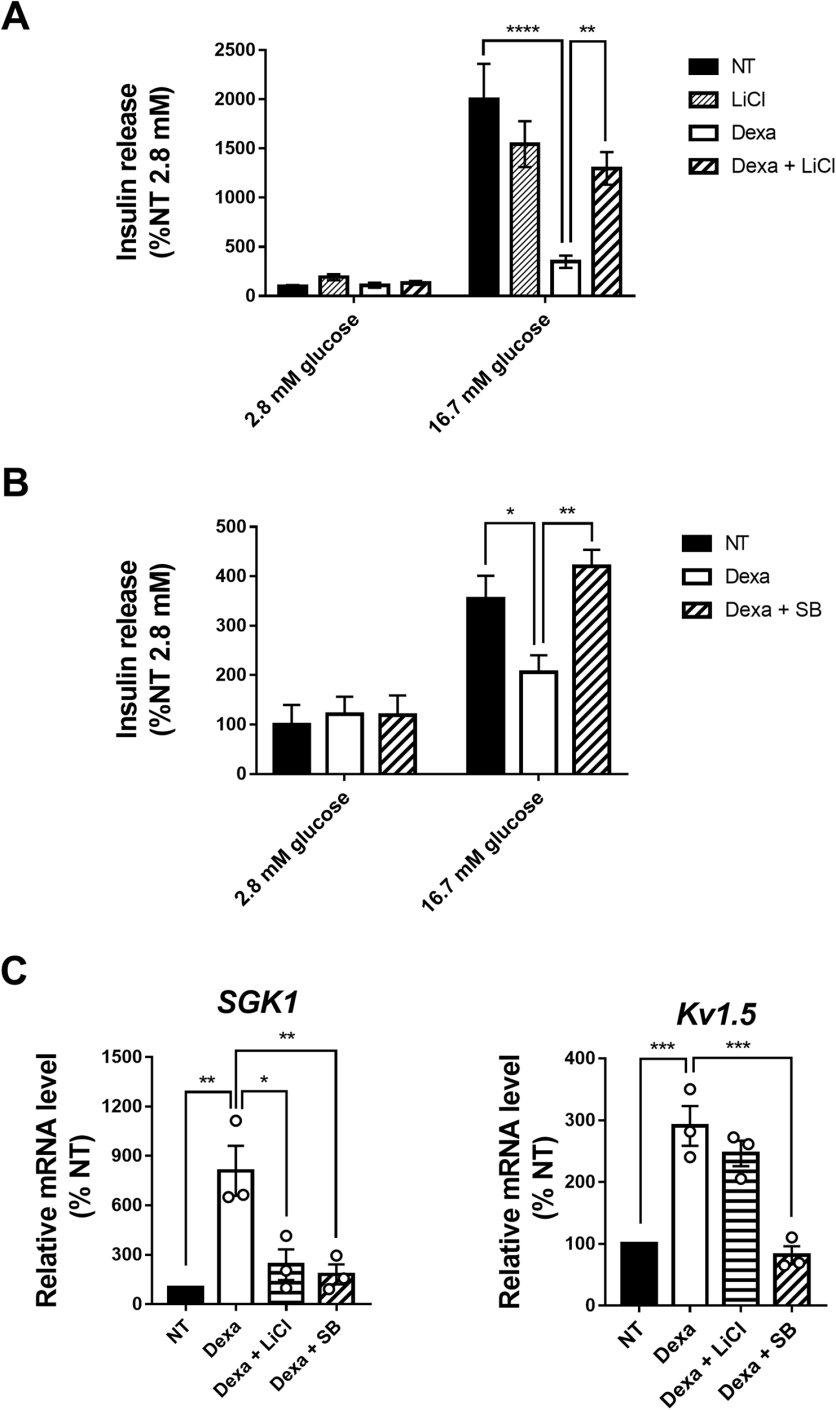
Treatment with LiCl or SB216763 improves insulin secretion in Dexa condition in rat islets. **A** Measurement of insulin secretion (*n* = 5) in rat islets after 24 h incubation with or without Dexamethasone (Dexa) and 27 h incubation with or without LiCl. Insulin secretion is assessed in medium containing 2.8 mM glucose or 16.7 mM glucose. Insulin in the medium was measured by ELISA and normalized to the DNA content. Results are expressed as percentage of NT 2.8 mM condition. **B** Measurement of insulin secretion (*n* = 5) in rat islets after 24 h incubation with or without Dexamethasone (Dexa) and 27 h incubation with or without SB216763 (SB). Insulin secretion is assessed in medium containing 2.8 mM glucose or 16.7 mM glucose. Insulin in the medium was measured by ELISA and normalized to the DNA content. Results are expressed as percentage of NT 2.8 mM condition. **C** Relative mRNA levels of *Sgk1* and *Kv1.5*, measured by qPCR (*n* = 3) in islets, after 24 h incubation with or without Dexamethasone (Dexa) and 27 h incubation with or without LiCl or SB216763 (SB). Cyclophilin A is used as housekeeping gene. Results are expressed as percentage of NT condition for each independent experiment. Results are shown as means ± S.E.M. *n* represents the number of independent experiments. *p < 0.05; **p < 0.01; ***p < 0.001; ****p < 0.0001.

GCs are known to impair β-cell secretory function [17, 18]. In our study, rat islets exposed to Dexa showed decreased insulin secretion in response to glucose, compared to untreated islets (Fig. 2A, B). Insulin secretion was restored in Dexa-treated islets following GSK3 inhibition with LiCl or SB216763 (Fig. 2A, B). Interestingly, the impairment of insulin secretion induced by Dexa treatment was associated with the upregulation of the expression of *Kv1.5*, a potassium channel which negatively regulates insulin secretion, and *SGK1*, which controls the activity of Kv1.5 (Fig. 2C). The inhibition of GSK3 partially reversed the upregulation of these factors.

### GC signaling does not strongly affect the expression of GSK3, neither its activity

**Fig. 3:**
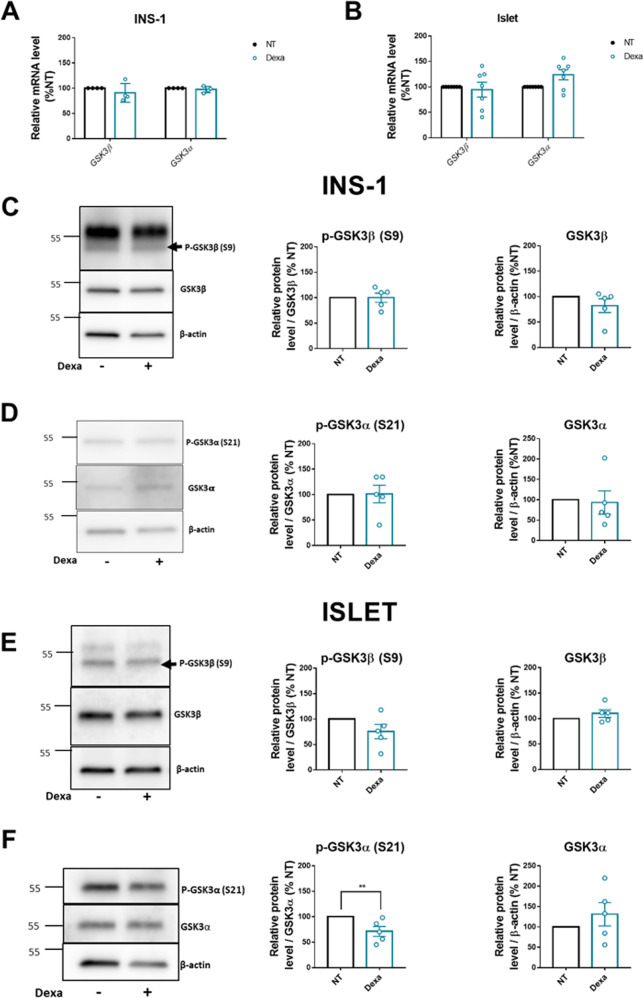
Dexa does not change GSK3α and GSK3β expression in INS-1 832/13 and in rat islets neither their activity. **A** Relative mRNA levels of *Gsk3α* and *Gsk3β*, measured by qPCR (*n* = 4) in INS-1 832/13, after 24 h incubation with or without Dexamethasone (Dexa). Results are expressed as percentage of NT condition for each independent experiment. **B** Relative mRNA levels of *GSK3α* and *GSK3β*, measured by qPCR (*n* = 7), in islets, after 24 h incubation with or without Dexamethasone (Dexa). Results are expressed as percentage of NT condition for each independent experiment. Cyclophilin A is used as housekeeping gene in **A** and **B**. **C** Representative Western Blot (left) and quantification (right) of P-GSK3β (S9) and GSK3β protein levels in INS-1 832/13 cells after 24 h incubation with or without Dexamethasone (Dexa) (*n* = 5). β-actin is used as loading control. Results are expressed as percentage of NT for each independent experiment. Black arrow shows the band corresponding to P-GSK3β (S9). **D** Representative Western Blot (left) and quantification (right) of P-GSK3α (S21) and GSK3α protein levels in INS-1 832/13 cells after 24 h incubation with or without Dexamethasone (Dexa) (*n* = 5). β-actin is used as loading control. Results are expressed as percentage of NT for each independent experiment. **E** Representative Western Blot (left) and quantification (right) of P-GSK3β (S9) and GSK3β protein levels in rat islets after 24 h incubation with or without Dexamethasone (Dexa) (*n* = 5). β-actin is used as loading control. Results are expressed as percentage of NT for each independent experiment. Black arrow shows the band corresponding to P-GSK3β (S9). **F** Representative Western Blot (left) and quantification (right) of P-GSK3α (S21) and GSK3α levels in rat islets after 24 h incubation with or without Dexamethasone (Dexa) (*n* = 5). β-actin is used as loading control. Results are expressed as percentage of NT for each independent experiment. Results are shown as means ± S.E.M. *n* represents the number of independent experiments. **p < 0.01.

Studies in other cell types have shown that GCs can regulate GSK3 activity [25]. Here, we investigated the effects of Dexa on both GSK3 expression and activity. First, we showed that treatment of INS-1 832/13 cells or primary islets with Dexa did not result in significant changes in mRNA (Fig. 3A, B) and protein levels of GSK3α or GSK3β (Fig. 3C–F), although in islets, the expression of GSK3α tended to increase slightly but not significantly (Fig. 3B).

GSK3 activity is regulated by phosphorylation. The phosphorylation of serine 9 (S9) in GSK3β and serine 21 (S21) in GSK3α are known to decrease their activity. Dexa treatment in INS-1 832/13 cells did not change the levels of phosphorylated S9 and S21 in GSK3β and GSK3α respectively (Fig. 3C, D). In islets, the phosphorylation of GSK3β was not significantly affected, while the phosphorylation of GSK3α was slightly but significantly decreased, reflecting an increased activity of this isoform (Fig. 3E, F).

### GSK3 participates to the nuclear localization of GR induced by Dexa

**Fig. 4:**
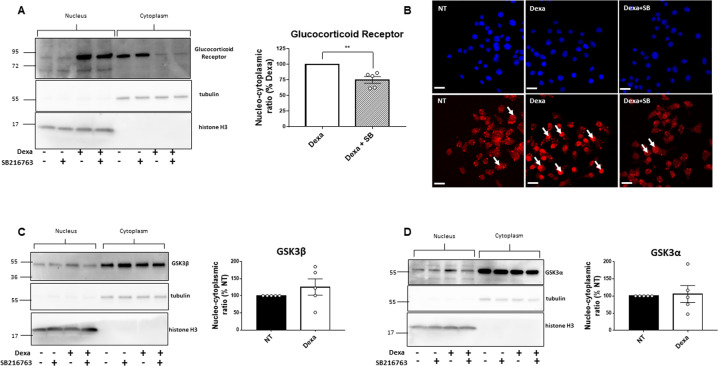
Treatment with SB216763 reduces the nuclear GR localization following Dexa treatment. **A** Representative Western Blot (left) and quantification (right) of nuclear and cytoplasmic GR protein levels in INS-1 832/13 after 3 h incubation with or without Dexamethasone (Dexa) and with or without SB216763 (SB) (*n* = 5). Tubulin is used as the marker of the cytoplasmic fraction. Histone H3 is used as the marker of the nuclear fraction. Results are expressed as the percentage of Dexa condition for each independent experiment. **B** Representative images showing DAPI (upper panel) and GR staining (lower panel) in INS-1 832/13 after 3 h incubation with or without Dexamethasone (Dexa) and 3 h with or without SB216763 (SB). White arrows show examples of nuclear staining of the GR. Scale bar: 20 μm. **C** Representative Western Blot (left) and quantification (right) of nuclear and cytoplasmic GSK3β protein level in INS-1 832/13 after 3 h incubation with or without Dexamethasone (Dexa) and with or without SB216763 (SB) (*n* = 5). Tubulin is used as the marker of the cytoplasmic fraction. Histone H3 is used as the marker of the nuclear fraction. Results are expressed as the percentage of NT condition for each independent experiment. **D** Representative Western Blot (left) and quantification (right) of nuclear and cytoplasmic GSK3α levels in INS-1 832/13 after 3 h incubation with or without Dexamethasone (Dexa) and with or without SB216763 (SB) (*n* = 5). Tubulin is used as the marker of the cytoplasmic fraction. Histone H3 is used as the marker of the nuclear fraction. Results are expressed as the percentage of NT condition for each independent experiment. Results are shown as means ± S.E.M. *n* represents the number of independent experiments. **p < 0.01.

GCs signaling implicates the nuclear localization of GR upon binding to GC. We performed cell fractionation experiments to separate different cellular compartments. Following 3 h of treatment with Dexa and/or SB216763, the cytoplasmic and nuclear fractions of INS-1 832/13 cells were separated, and the purity of fractions was confirmed by the immuno-detection of nuclear marker histone H3 and cytoplasmic marker tubulin, as shown in Fig. 4A.

As expected, Dexa treatment strongly induced the nuclear translocation of the GR, as shown by immuno-detection of the GR in the nuclear fraction by western blot, and by immunofluorescent staining (Fig. 4A, B). Treatment with SB216763 slightly but reproducibly reduced the nuclear level of GR protein in Dexa-treated cells, as shown by the decreased nucleo-cytoplasmic ratio of GR (Fig. 4A). Immunofluorescence staining of GR confirmed these results (Fig. 4B). Moreover, GSK3α and GSK3β localization were monitored and we found no changes in their sub-cellular localization following Dexa treatment (Fig. 4C, D). However, it should be noted that there was an important heterogeneity in this parameter between the independent experiments.

### GSK3 inhibition decreases the expression of GR in INS-1 832/13 cells as well as in rat and human islets

**Fig. 5:**
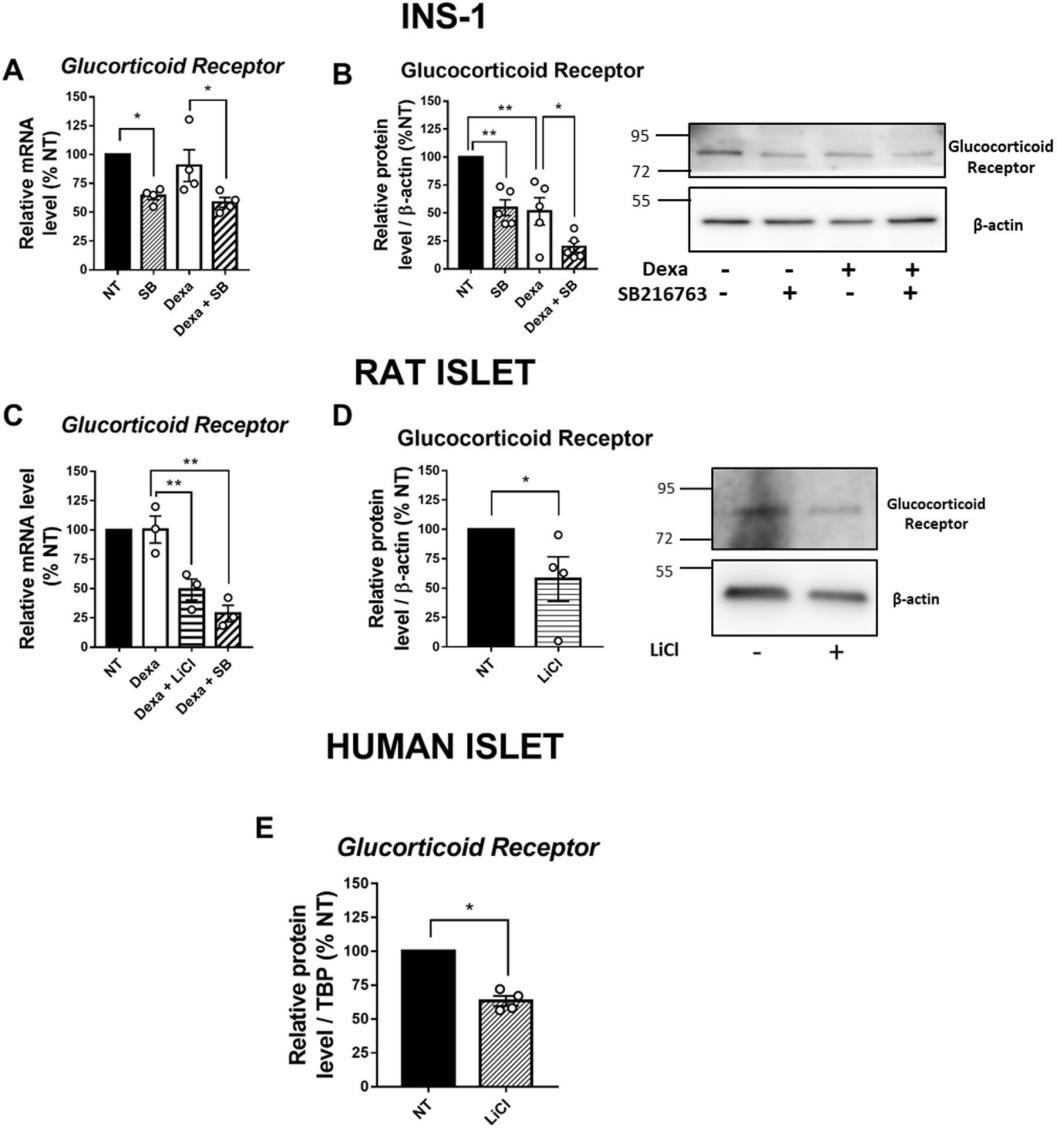
Treatment with LiCl or SB216763 reduces the GR expression in INS-1 832/13 cells as well as in rat and human islets. **A** Relative mRNA level of *GR*, measured by qPCR (*n* = 4), in INS-1 832/13, after 24 h treatment with or without Dexamethasone (Dexa) and 27 h with or without SB216763 (SB). Results are expressed as percentage of NT condition for each independent experiment. **B** Representative Western Blot (right) and quantification (left) of GR protein level in INS-1 832/13 after 24 h incubation with or without Dexamethasone (Dexa) and 27 h incubation with or without SB216763 (SB) (*n* = 5). β-actin is used as loading control. Results are expressed as percentage of NT condition for each independent experiment. **C** Relative mRNA level of *GR*, measured by qPCR (*n* = 3) in rat islets, after 24 h incubation with or without Dexamethasone (Dexa) and 27 h incubation with or without LiCl or SB216763. Results are expressed as percentage of NT condition for each independent experiment. **D** Representative Western Blot (right) and quantification (left) of GR protein level in rat islets after 24 h incubation with or without Dexamethasone (Dexa) and 27 h incubation with or without LiCl (*n* = 4). β-actin is used as loading control. Results are expressed as percentage of NT condition for each independent experiment. **E** Relative mRNA level of human *GR*, measured by qPCR (*n* = 4), in human islets, after 24 h treatment with or without LiCl. Results are expressed as percentage of NT condition for each independent experiment. Cyclophilin A is used as housekeeping gene in figure **A** and **C**. TBP is used as housekeeping gene in figure **E**.

In addition to the evaluation of GSK3 expression following Dexa treatment described above, we assessed the mRNA and protein levels of GR upon GSK3 inhibition. We found that SB216763-treated INS-1832/13 cells exhibited decreased levels of GR mRNAs and proteins (Fig. 5A, B). Dexa-treated INS-1 832/13 cells also showed decreased GR protein level, likely reflecting an adaptive mechanism [26]. However, a cumulative effect was observed in Dexa + SB216763 condition in comparison to Dexa alone, pointing to a proper implication of GSK3 in this phenomenon. Similar to what we found in INS-1 832/13 cells, GSK3 inhibition significantly reduced GR expression in islets at mRNA (Fig. 5C) and protein levels (Fig. 5D). Moreover, we assessed the mRNA level of GR in human islets treated or not with LiCl. As found in rat islets, the mRNA level of GR was significantly decreased in human islets upon GSK3 inhibition with LiCl (Fig. 5E).

### GSK3β has a major role in GR expression and its downregulation is sufficient to abrogate GC-induced β-cell death

**Fig. 6:**
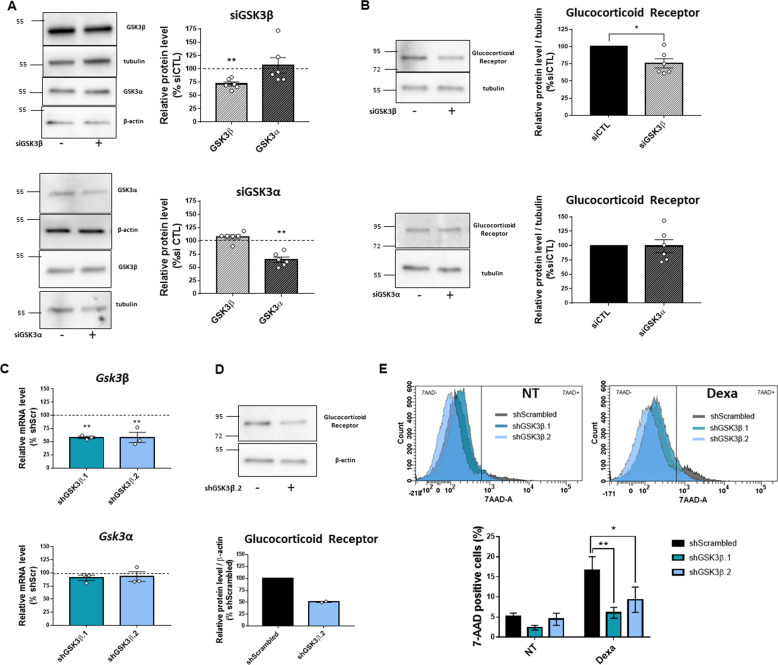
GSK3β knock-down is sufficient to decrease GR expression and GC-induced β-cell death. **A** Representative Western Blot (left) and quantification (right) of GSK3β and GSK3α levels in INS-1 832/13 after 24 h incubation with GSK3β siRNA (upper panel, *n* = 6) or with GSK3α siRNA (lower panel, *n* = 6). β-actin is used as loading control for GSK3α and tubulin is used as loading control for GSK3β. Results are expressed as percentage of siCTL condition, represented by dashed line, for each independent experiment. **B** Representative Western Blot (left) and quantification (right) of GR protein level in INS-1 832/13 after 24 h incubation with GSK3β siRNA (upper panel, *n* = 6), or with GSK3α siRNA (lower panel, *n* = 6). Tubulin is used as loading control. Results are expressed as percentage of siCTL condition for each independent experiment. **C** Relative mRNA level of GSK3β (upper panel) and GSK3α (lower panel), measured by qPCR, in INS-1 832/13, after infection with lentivirus coding for scrambled shRNA (shScr), GSK3β.1 shRNA (shGSK3β.1) or GSK3β.2 shRNA (shGSK3β.2) (*n* = 3). Results are expressed as percentage of scrambled shRNA condition, represented by dashed line, for each independent experiment. **D** Representative Western-blot (upper panel) and quantification (lower panel) of GR protein level in GFP sorted INS-1 832/13 after infection with lentivirus coding for scrambled shRNA or GSK3β.2 shRNA (*n* = 2). β-actin is used as loading control. Results are expressed as percentage of scrambled shRNA condition for each independent experiment. **E** Cell death reported by 7-AAD incorporation measured by flow cytometry, and quantification (*n* = 5), after infection of INS-1 832/13 cells with lentivirus coding for scrambled shRNA, GSK3β.1 shRNA or GSK3β.2 shRNA, and 24 h after incubation with (right panel) or without Dexamethasone (Dexa) (left panel). Cyclophilin A is used as housekeeping gene in figure **C**. Results are shown as means ± S.E.M. *n* represents the number of independent experiments. *p < 0.05; **p < 0.01.

Because pharmacological inhibitors used in this study (LiCl and SB216763) inhibit both GSK3α and GSK3β isoforms, we used genetic approaches to further dissect the proper role of each isoform in the regulation of the GR expression. Using isoform-specific siRNAs, we knocked down GSK3α or GSK3β in INS-1 832/13. First, we validated by western blot the efficacy of siRNAs in reducing each targeted isoform (Fig. 6A). Furthermore, to rule out a possible compensatory expression between isoforms, we monitored the levels of the non-targeted isoform following the use of the respective GSK3 isoform-specific siRNAs (Fig. 6A). Following these validation steps, we monitered GR protein level by western-blotting. We found that the down-regulation of GSK3β, but not GSK3α, is sufficient to reduce GR protein levels in INS-1 832/13 cells (Fig. 6B), to a similar extent as that observed with pharmacological inhibitors. Thereafter, we focused on GSK3β and created a most efficient downregulation system, using shRNA strategy. We used GSK3β-specific shRNA expression vectors (2 different sequences) and produced pGFP-C lentiviral transduction particles. After viral infection of INS-1 832/13 cells, we validated their efficacy by showing that both GSK3β-specific shRNA sequences efficiently decreased mRNA levels of GSK3β, without alterating the levels of GSK3α mRNAs (Fig. 6C). The efficiency of lentiviral infection does not reach 100% in INS-1 832/13 cells. To precisely evaluate the role of GSK3β in the regulation of GR expression, we decided to work on cells with effective GSK3β knockdown. We GFP-sorted shGSK3β infected cells, and performed immunoblotting for GR in the GFP-positive population. We showed that in these cells, the expression of GR was reduced by 50% (Fig. 6D).

Next, we carried out additional experiments to determine whether GSK3β knockdown can oppose the GC-induced cell death in INS-1 832/13 cells. We showed that in GFP-positive cells, which represent cells expressing GSK3β shRNAs, the percentage of 7-AAD positive cells following Dexa treatment was significantly lower compared to the percentage found in Dexa-treated cells infected with scrambled shRNAs, suggesting that GSK3β mediates the pro-apoptotic effects of Dexa in β-cells (Fig. 6E). Interestingly, the knockdown of GSK3β alone produced the same protective effects against GC-induced cell death, as those observed with pharmacological inhibitors (Fig. 1A, B), supporting yet the specific implication of the β isoform in this process.

## Discussion

Diabetogenic effects of GCs are largely documented [14, 27–30]. Chronically high levels of GCs, such as those observed in patients suffering from Cushing’s syndrome [31], or in case of prolonged administration of GCs for therapeutic purposes, result in metabolic disturbances which are in part due to their deleterious effects on pancreatic β-cells. GCs are described to alter both β-cell survival and secretory function in humans and rodents, in most studies [15, 17–19, 32–35], although a limited number of studies report the opposite [36, 37]. While the impairment of β-cell function and survival by GCs has been extensively documented, to date, there is a dearth of information abouth the crosstalk between GCs signaling and GSK3 in this cell type. Only one study has investigated the implication of GSK3 in the GC-induced β-cell apoptosis [38]. However, this study has not been conducted in islets but only in a non-specified clone of INS-1 cell line and did not address the implication of GSK3 in insulin secretion, neither the proper contribution of GSK3β and GSK3α. In the present study, we unraveled for the first time the mechanisms underlying the implication of GSK3 in the adverse effects of GCs on β-cells, and provided evidence for the relevance of targeting GSK3 as a means to preserve both β-cell survival and secretory function, in the context of GC excess. First, using pharmacological and genetic tools, we showed that the pro-apoptotic effects of GCs in β-cells can be reversed by GSK3 inhibition. The underlying mechanisms of the GC-mediated β-cell apoptosis are only partially known. It is generally admitted that this process takes place mostly through the activation of the intrinsic apoptotic pathway in β-cells [19, 39, 40], although one study showed recently an effect on the extrinsic pathway [41]. We therefore investigated the expression of members of bcl-2 family of proteins, namely Bim and Bcl-2, which are key players in the GC-induced intrinsic apoptotic pathway [19, 40]. We showed that GSK3 is implicated in the Dexa-induced upregulation of Bim, but not in the downregulation of Bcl-2 induced by Dexa. These results are in line with a previous study in a lymphoma cell line, where only the upregulation of Bim induced by GCs was dependent on GSK3 [42]. This could be explained by the fact that, as reported by Rogatsky and colleagues, GSK3 controls the transactivation activity of GR in rodent cells, via the phosphorylation of Thr171, whereas it does not impact the transrepression activity of the GR on AP-1 [43].

The inhibitory effect of GCs on insulin secretion has been reported both in rodent and human islets [15, 17]. Some of these effects are mediated by the induction of the expression of *SGK1* and *Kv1.5*, which control the K^+^ outflow in β-cells and therefore reduce insulin secretion in vitro in INS-1 cells and in vivo [33, 44]. In our study, we confirmed the effects of Dexa on the impairment of the glucose-induced insulin secretion and the stimulation of *SGK1* and *Kv1.5* expression in rat islets. Importantly, we show for the first time that pharmacological inhibition of GSK3 by either LiCl or SB216763 nearly normalized the glucose-induced insulin secretion in Dexa-treated islets. Furthermore, associated with the recovery of normal insulin secretion, the upregulation of *SGK1* and *Kv1.5* by Dexa was abrogated by GSK3 inhibitors. The mechanisms underlying the interactions between GSK3 and the GCs pathway have been investigated in several cell systems [22, 23, 43, 45]. However, despite the central roles of both GSK3 [46–50] and GCs [15, 17, 19, 51] in β-cell growth, survival and secretory function, their crosstalk in these cells has not been addressed. To the best of our knowledge, the present study is the first to uncover several aspects of the complex interplay between GSK3 and the GC pathway in β-cells. First, since GR acts as a regulator of gene transcription, we asked whether the activation of GR by Dexa could affect the expression of GSK3β and GSK3α. We found that the expression of neither of GSK3 isoforms was affected by Dexa treatment in terms of mRNAs nor proteins. As an additional mechanism, GSK3β activity has been reported to be enhanced by GCs through the reduction of the phosphorylation of Ser9 residue of GSK3β [25, 38, 52–55], hence increasing its activity. Since GSK3 is a negative regulator of growth and survival in β-cells [46–50], we hypothesized that GCs pro-apoptotic effects could be mediated partly by the stimulation of GSK3 activity upon GC treatment. Here, we showed that GSK3 phosphorylation status was not affected by Dexa in INS-1 832/13 cells. However, in rat islets, while GSK3β phosphorylation on Ser9 was not affected, we found a slight but significant decrease of GSK3α phosphorylation on Ser21, suggestive of its increased activity induced by Dexa, although this mechanism is likely to play a minor role, if any, given the small difference observed between Dexa group and the untreated group.

Most cellular effects of GCs are mediated by the nuclear translocation of GR upon binding of its ligands. Previous studies have shown that GSK3 participates to the GR nuclear localization in human osteoblasts [23] and in human lymphocytes [22]. In our study, we showed for the first time in β-cells, that the inhibition of GSK3 slightly but reproducibly reduced GR’s nuclear localization. However, this mechanism could only partly account for the mitigation of GCs effects by GSK3 inhibitors in β-cells. Importantly, on the other hand, we consistently found that both in rat and human islets, as well as in INS-1 832/13 cells, GSK3 inhibition resulted in a significant decrease of the expression of GR, both at mRNA and protein levels. Interestingly, this mechanism could be β-cell specific since in others cell system GSK3 inhibition did not result in downregulation of GR [22, 23, 43]. These are novel and important findings, which have not been reported elsewhere. In light of these findings, we could speculate that one of the multiple roles of GSK3 in β-cells could be the maintenance of GR at an optimal level, thus contributing, in addition to other mechanisms, to the deleterious effects of GCs on both β-cells survival and insulin secretion. This unexpected role of GSK3 in β-cells supports its relevance as a target to alleviate the side effects of GCs. The mechanisms through which GSK3 regulates the expression of GR are not known. It could be speculated that GSK3 would indirectly modulate the transcription of GR gene, and/or the stability of GR mRNA and/or protein in the cell, known to be influenced by its phosphorylation status [56].

Finally, the other major and novel finding of our study was the unraveling of the respective contribution of GSK3 isoforms α and β, to the mediation of pro-apoptotic effects of GCs in β-cells. Only few studies in other cells systems have investigated the isoform-specific interaction of GSK3 with the GC pathway, and they led to discordant conclusions [22, 45]. In the present study, through isoform-specific genetic downregulation, we showed for the first time that the knockdown of GSK3β isoform, and not that of GSK3α, led to a significant decreased expression of GR in β-cells. Moreover, GSK3β knockdown alone resulted in the abrogation of the pro-apoptotic effects of Dexa, to the same extent as the effects of the pharmacological inhibitors. While the contribution of GSK3α cannot be completely ruled out based on our data, we propose GSK3β as the main GSK3 isoform implicated in the GC-induced cell death in β-cells.

To sum up, this study provides solid evidence for the central role of GSK3 in promoting the diabetogenic effects of GCs in β-cells, both in term of survival and insulin secretion. Importantly, we unraveled for the first time some of the mechanisms underlying GR-GSK3 crosstalk in β-cells, and identified GSK3β as the main isoform contributing to GC-induced β-cell death.

GCs are widely prescribed for their anti-inflammatory and immunosuppressive properties as treatment for a variety of diseases, including diseases with no alternative therapies. Given the serious diabetogenic side effects of GCs, it is crucial to find new strategies to circumvent the adverse effects of elevated levels of endogenous or exogenous GCs, on organs involved in the regulation of glucose metabolism.

Our work has contributed to the identification of GSK3 as a viable target to mitigate GC deleterious effects in pancreatic β-cells. These novel findings indicate that in susceptible individuals with pre-diabetic condition, GC therapy may become significantly safer, by combining GCs with GSK3 inhibitors as an adjunct treatment, which interestingly has been shown to have powerfull anti-inflammatory effects in chronic inflammatory diseases [46, 57–60].

## Materials and methods

### Animals

Animals used in this study were male Wistar rats from our local colony. Animal housing and breeding and relevant procedures were conducted in accordance with the European Community Council directives (2010/63/UE) and approved by the institutional Animal Care and Use Ethical Committee of the Paris-Diderot University (Agreement B-75-13-17). Animal were housed in controlled environment (temperature = 20 ± 1 °C; humidity = 60%) with food and water *ad libitum* on 12 h light/dark cycle. After weaning, animals were fed with a standard chow diet (diet D113, SAFE, Augy, France).

### Rat islet isolation

Rat islets were isolated from 10–12 weeks old male Wistar rats. Pancreatic islets were isolated by Liberase (Roche, Boulogne Billancourt, France, ref. 05401020001) digestion (37 °C, 15 min) and Histopaque®1077 (Sigma, Saint Quentin Fallavie, France, ref. 10771) gradient purification as previously described [61]. Islets were hand-picked under a stereomicroscope and divided into different batches.

### Islet and β-cell culture and treatments

Batches of 150 freshly isolated islets were stabilized in RPMI 1640 + Glutamax medium (Gibco, Illkirch, France, ref. 61870036) with 10% Fetal Calf Serum (FCS) (Eurobio, Les Ulis, France, ref. CVFSVF00–01), 1% Penicillin-Streptomycin (Sigma, Saint Quentin Fallavie, France, ref. P4333), 1% Hepes (Sigma, Saint Quentin Fallavie, France, ref. H0887), 1% Sodium pyruvate (Sigma, Saint Quentin Fallavie, France, ref. S8636) during 36 h. Islets were then pre-treated or not with 10 mM LiCl (Sigma, Saint Quentin Fallavie, France, ref. L7026) or with 20 μM SB216763 (Sigma, Saint Quentin Fallavie, France, ref. S3442) during 3 h. Next, Dexamethasone (Sigma, Saint Quentin Fallavie, France, ref. D4602) at the concentration of 100 nM was added or not to the medium and incubated for 24 h. Islets were then processed for histology, insulin secretion measurement, real-time PCR or Western blot analysis.

Rat insulinoma INS-1 832/13 cells were grown in RPMI 1640 + Glutamax medium with 10% Fetal Calf Serum, 1% Penicillin-Streptomycin, 1% Hepes, 1% Sodium Pyruvate, 50 μM β-mercaptoethanol (Gibco, Illkirch, France, ref. 3150-010). INS-1 832/13 cells were treated or not with 20 μM SB216763 and 100 nM Dexamethasone according to the protocol described above. For immunofluorescence analysis and cellular fractionation, cells were subjected to SB216763 and Dexamethasone treatment during only 3 h, in FCS-free medium.

### Human islets

Human islets were isolated from four brain-dead non-diabetic donors with prior consent for research use at Saint-Louis Hospital (Paris, France) according to a modified version of the automated method [62, 63]. The isolated islets were washed and cultured in CMRL 1066 medium (Sigma, Saint Quentin Fallavie, France, ref. CR1136), which contained 5.6 mM glucose, 25 mM Hepes, 2 mM glutamine, 100 U/mL penicillin, 100 mg/mL streptomycin, and 10% fetal calf serum. After reception, human islets were cultured for an additional 48 h for recovery in our laboratory. Then, they were treated or not with 10 mM LiCl during 24 h, and processed for real-time PCR.

### Cell transfection

INS-1 832/13 cells have been transfected with 5 nM siRNA, using the protocol Fast-Forward Transfection with HiPerFect Transfection Reagent (Qiagen, Courtaboeuf, France) following the manufacturer instructions, in antibiotics and serum-free OPTI-MEM + Glutamax medium (Gibco, Illkirch, France, ref. 51985-026), on 5 × 10^5^ cells. Following transfection, cells were cultured in OPTI-MEM supplemented with 10% Fetal Calf Serum, 1% Penicillin-Streptomycin, 1% Hepes, 1% Sodium pyruvate, 50 µM β-mercaptoethanol. The following siRNAs have been used: Control siRNA (Qiagen, Germantown, Maryland, USA, ref. 1022076), siGSK3β-Flexitude_3 (Qiagen, Germantown, Maryland, USA, ref. SI03056879), siGSK3α-Flexitube_2 (Qiagen, Germantown, Mariland, USA, ref. SI03080105). siRNAs target sequences are provided in Table 1.

**Table 1:** List of rat siRNAs sequences.

Gene targeted	Sequence targeted
siCTL duplex	5′-AATTCTCCGAACGTGTCACGT-3′
siGSK3α	5′-ACGTCGATTCACACCATCCAA-3′
siGSK3β	5′-CCCAAATTATACAGAATTCAA-3′

### Lentiviral production, titration and cell infection

We used two GSK3β shRNA constructs (Origene, Herford, Germany, ref TL711670#A and TL711670#C) and a scrambled shRNA (Origene, Herford, Germany, ref TR30021). The cassettes were inserted in a lentiviral GFP vector. shRNA sequences are reported in Table 2. Lentiviral particles were produced by transient transfection of HEK293T cells (ATCC® CRL-3216™) using calcium phosphate with the packaging plasmids pMD2.G (Addgene ref 12259) and psPAX2 (Addgene ref 12260) to form the viral particles, together with a rat shRNA-GFP lentiviral construct.

**Table 2:** List of rat shRNA sequences.

shRNA	Sequence
shScrambled	5′-GCACTACCAGAGCTAACTCAGATAGTACT-3′
shGSK3β.1	5′-ACCGTGGACAGACCAATAACGCCGCTTCT-3′
shGSK3β.2	5′-AGCCTCAGATACTAATGCTGGAGACCGTG-3′

The culture medium was collected 48 h after transfection of HEK293T and filtered through 0.45 µm pores. Lentiviral particles were concentrated by ultracentrifugation at 25,000 r.p.m. for 2 h and then resuspended in 200 µL PBS. For each construct, the lentiviral titer was determined by infection of HEK293T cells and FACS analysis.

2 × 10^5^ INS-1 832/13 cells were infected with lentiviral particles containing scrambled shRNA-GFP, GSK3β.1 shRNA-GFP, or GSK3β.2 shRNA-GFP construct (MOI of 10). Infected cells were treated or not with 100 nM Dexamethasone during 24 h and processed for real-time PCR, western blotting or flow cytometry analysis.

### Flow cytometry

INS-1 832/13 cell death has been assessed by 7-AAD (BD Biosciences, Le Pont de Claix, France, ref. 28025013) incorporation or by Annexin V-FITC incorporation (Biolegend, San Diego, California, USA, ref. 640922) assay according to the manufacturer’s instructions and quantified on a FACS ARIA II flow cytometer (BD Biosciences, Le Pont de Claix, France).

### Real-time PCR

Total RNA was extracted from rat islets, human islets or INS-1 832/13 cells using the RNeasy mini kit (Qiagen, Courtaboeuf, France, ref. 74104). cDNA of each RNA sample was synthesized with Moloney murine leukemia virus (M-MLV) reverse transcriptase kit (Invitrogen, Illkirch, France, ref. 28025013). Real-time quantitative PCR amplification reactions were carried out in a LightCycler 480 detection system (Roche, Boulogne-Billancourt, France) using the LightCycler SYBR Green 480 kit (Roche, Boulogne-Billancourt, France, ref. 04887352001). The primer sets were designed using OLIGO7 and are described in Table 3. All reactions were run in duplicate. Cyclophilin A were chosed as housekeeping gene for rat and TBP were chosed as housekeeping gene for human islets, as described in [44]. The PCR program was set as follows: 95 °C 10 min, followed by 40 cycles at 95 °C 10 s, 60 °C 10 s and 72 °C for 10 s.

**Table 3:** List of primers.

Gene	Forward	Reverse
*Bcl-2*	5′-CGGTGGTGGAGGAACTCTTCA-3′	5′- CTGGGGCCATATAGTTCCACAA-3′
*Bim*	5′- AAGTCAACACAAACCCCAAGTCC-3′	5′- CCTCCTCGTGTAAGTCTCATTGAAC-3′
*Chop*	5′- CAAGCACCTCCCAAAGCCCTCG-3′	5′- CCTTCTCCTTCATGCGCTGT-3′
*Cyclophilin A*	5′- AACCCACCGTGTTCTTC-3′	5′- TGCCTTCTTTCACCTTCCC-3′
*Glucocorticoid Receptor*	5′-TTTCCCAAAACTCACTCGGAT-3′	5′-TACAATTTCACACTGCCTCCGTT-3′
*Gsk3α*	5′-GGCCCTGCTACCCTCACCTCG-3′	5′- CCCTTTCCAGCCCACTTGAGCCT-3′
*Gsk3β*	5′- TATAGTCGAGCCAAGCAGACACTCC-3′	5′-TGCCCTGTAGTACCGAGAACA-3′
*Kv1.5*	5′-ACTTCGCAGAGGCAGACAATCA-3′	5′-GGTTGCCTTGTTCTTCCTTCAG-3′
*Nox4*	5′-AGAAAGATTCCGAGATTTACTACTGC-3′	5′- AAACGGAGTGACCCCAATGC-3′
*Sgk1*	5′-AAACCTATTGAAACGGTCTTGC-3′	5′-ACGGCTCTGACTGACAAC-3′
*Sod1*	5′- TGGTCCACGAGAAACAAGATG-3′	5′- AATCCCAATCACACCACAAGCCAA-3′
*Sod2*	5′- GGCCAAGGGAGATGTTACAA-3′	5′- GACCCAAAGTCACGCTTG-3′
*Human Glucocorticoid Receptor*	5′-GAGCAATTCCAGTTTCACCTAAGTC-3′	5′-AAATCTCACTGAAGGCTACCAT-3′
*Human TBP*	5′-GCACCACTCCACTGTATCCC-3′	5′-ATGATTACCGCAGCAAACCG-3′

### Cell death detection

For immunohistochemical studies, following the treatments described above, islets from each group were hand-picked, fixed in aqueous Bouin’s solution for 1 h, embedded in agarose (Sigma, Saint Quentin Fallavie, France, ref. A6013) and then in paraplast (Leica, Nanterre, France, ref. 39602004). Blocs were serially sectioned (5 μm thickness). Cell death was assessed by the TUNEL apoptosis detection kit (Sigma, Saint Quentin Fallavie, France, ref. S7101) on islet sections, according to the manufacture’s protocol, with the modification of TdT enzyme concentration which was further diluted (1/10) to adapt to our experimental conditions. Quantification was made using a computer-assisted image analysis system Histolab 5.14 software (Microvision Instruments, Lisses, France). At least 20 islets were counted per experimental condition.

### Measurement of glucose-induced insulin secretion

Freshly isolated Wistar islets were stabilized in RPMI1640 + Glutamax complete medium during 36 h, then transferred into a RPMI 1640 medium containing or not 10 mM LiCl or 20 µM SB216763 for pre-treatment. After 3 h pre-treatment, 100 nM Dexamethasone was added or not to the media for 24 h. Following 24 h incubation, batches of 10 islets from each group were pre-incubated for 1 h in a Krebs- Ringer/bicarbonate/Hepes buffer (115 mM NaCl, 5 mM KCl, 24 mM NaHCO_3_, 1 mM CaCl_2_, 1 mM MgCl_2_) containing 0.2% fatty-acid-free BSA (Roche, Boulogne-Billancourt, France, ref. 10775835001) and 2.8 mM glucose, followed by 1 h incubation in 2.8 or 16.7 mM glucose, to measure glucose-induced insulin secretion. Insulin released in the supernatant was measured by ELISA (Alpco Diagnostics, Salem, Massachusetts, USA, ref. 80-INSRTU-E01), and normalized to islet DNA content.

### Cellular fractionation

INS-1 832/13 cells were cultured for 3 h in serum-free RPMI1640 + Glutamax with or without SB216763 20 µM, and with or without Dexamethasone 100 nM. Cells were resuspended in SF buffer (250 mM Sucrose, 20 mM HEPES, 10 mM KCl, 1,5 mM MgCl_2_, 1 mM EDTA, 1 mM EGTA) supplemented extemporaneously with 1 mM DTT, 4% Protease Inhibitor Cocktail (Sigma, Saint Quentin Fallavie, France, ref. P5726) and 5 mM spermine (Sigma, Saint Quentin Fallavie, France, ref. S3256) and spermidine (Sigma, Saint Quentin Fallavie, France, ref. S2626). After 30 min agitation in SF, cells were centrifuged at 720 g for 10 min to separate non-lysed cells, nuclei and cytoplasm. The pellet containing nuclei were washed in SF and resuspended in NL buffer (50 mM Tris HCl pH = 8, 150 mM NaCl, 1% NP-40, 0.5% sodium deoxycholate, 0.1% SDS, 4% Protease Cocktail Inhibitor). The supernatant containing cytoplasm were washed to remove traces of nuclei and were ultracentrifugated at 100 000 g for 1 h to separate cytoplasm and membrane fractions. Western blot analysis were performed in nuclear and cytoplasmic fractions.

### Western blot analysis

Western blot analysis were performed on INS-1 832/13 or rat islets, lysed in RIPA buffer (Cell Signaling, Saint-Cyr, France, ref. 9806) with 4% protease inhibitor cocktail PIC, PIC2, PIC3 (Sigma, Saint Quentin Fallavie, France, ref. P5726). Fifteen µg protein were subjected to SDS-PAGE and transferred on a PVDF membrane (Sigma, Saint Quentin Fallavie, France, ref. IPVH09120). After blocking with 5% milk or 5% fatty acid free BSA, the membrane was incubated with corresponding primary antibody: anti-Bim (Cell Signaling, Saint-Cyr, France, ref. 2819), anti-Bcl-2 (eBiosciences, Illkirch, France, ref. 14-6992-82), anti-pSer9GSK3β (Cell Signaling, Saint-Cyr, France, ref. 5558), anti-GSK3β (Cell Signaling, Saint-Cyr, France, ref. 9315), anti-pSer21GSK3α (Cell Signaling, Saint-Cyr, France, ref. 9316), anti-GSK3α (Cell Signaling, Saint-Cyr, France, ref. 9338), anti-GR (Cell Signaling, Saint-Cyr, France, ref. 12041), anti-β-actin (Sigma, Saint Quentin Fallavie, France, ref. 2709), anti-Histone H3 (Cell Signaling, Saint-Cyr, France, ref. 4499), or anti-α/β-tubulin (Cell Signaling, Saint-Cyr, France, ref. 2148). The addition of appropriate horseradish peroxidase-conjugated secondary antibody (Jackson Labs, Las Vegas, USA) allowed to visualize the reaction using a chimioluminescence system with ECL Prime (Amersham, Les Ulis, France, ref 29018903). Membranes were then stripped using Re-Blot solution (Millipore, Molsheim, France, ref 2504) and then re-probed with appropriate antibody. The results were analyzed and quantified by scanning densitometry using FIJI®.

### Immunofluorescence

Following 3 h incubation with the different treatments described above, INS-1 832/13 cells were fixed in a solution of 4% formaldehyde (Sigma, France, ref. F8775). Then, cells were permeabilized with Triton 0.2% for 20 min, and incubated with 10% goat serum for 1 h. Cells were then incubated with anti-GR antibody overnight at 4 °C (Cell Signaling, France, ref. 12041) followed by incubation with anti-rabbit fluorescent secondary antibody for 1 h at room temperature. Finally, cells were covered with mounting medium containing DAPI. Images were acquired using confocal microscopy Zeiss LSM 700.

### Data and statistical analysis

Results are expressed as means ± S.E.M. In the legends, “*n*” is the number of independent experiments performed. When it was possible, the experimental groups were blinded to the investigator that performed the quantification/analyses. For each data set, the outliers were identified by the appropriate statistical test. The statistical significances between means were assessed by Mann–Whitney test (two groups), or by One-Way or Two-Way ANOVA followed by Tukey’s or Dunnett’s post-hoc analysis when more than two conditions were compared, using Graph-Pad Prism 7 software. *P*-values are indicated in graph as followed: *p < 0.05; **p < 0.01; ***p < 0.001; ****p < 0.0001.

## Supplementary information

Supplemental Figure 1

Supplemental Figure 2

Reproducibility checklist

## Data Availability

The raw data supporting the conclusions of this manuscript will be made available by the authors, without undue reservation, to any qualified researcher.
