# Supplementary figures and images for: Underlying mechanisms of glucocorticoid-induced β-cell death and dysfunction: a new role for glycogen synthase kinase 3
Source: Cell Death Dis. 2021 Dec 7;12(12):1136. doi: 10.1038/s41419-021-04419-8 (PMC8651641; doi:10.1038/s41419-021-04419-8)

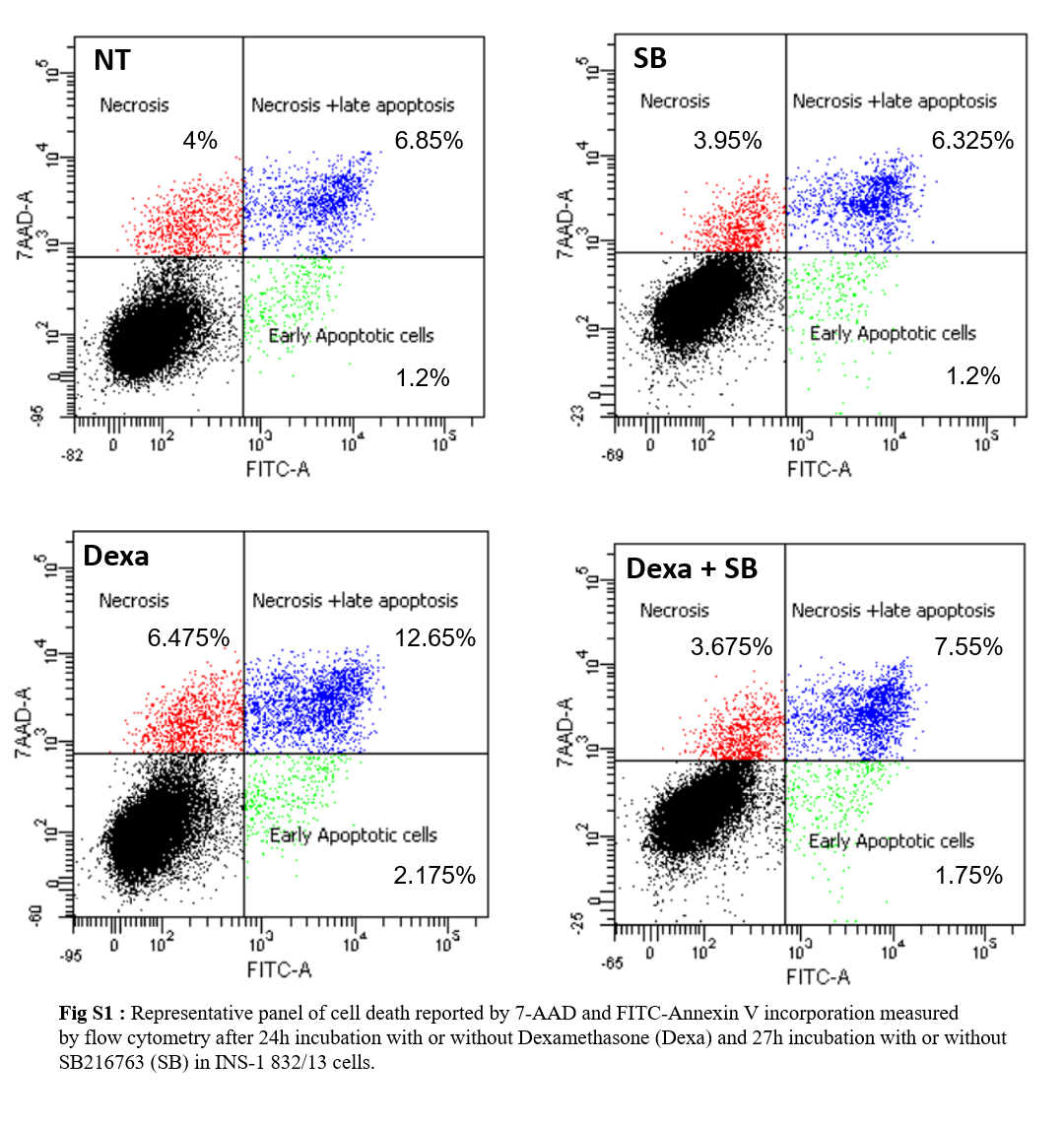

Supplement: Supplementary file 1 — Supplemental Figure 1 [file 41419_2021_4419_MOESM1_ESM.tif]

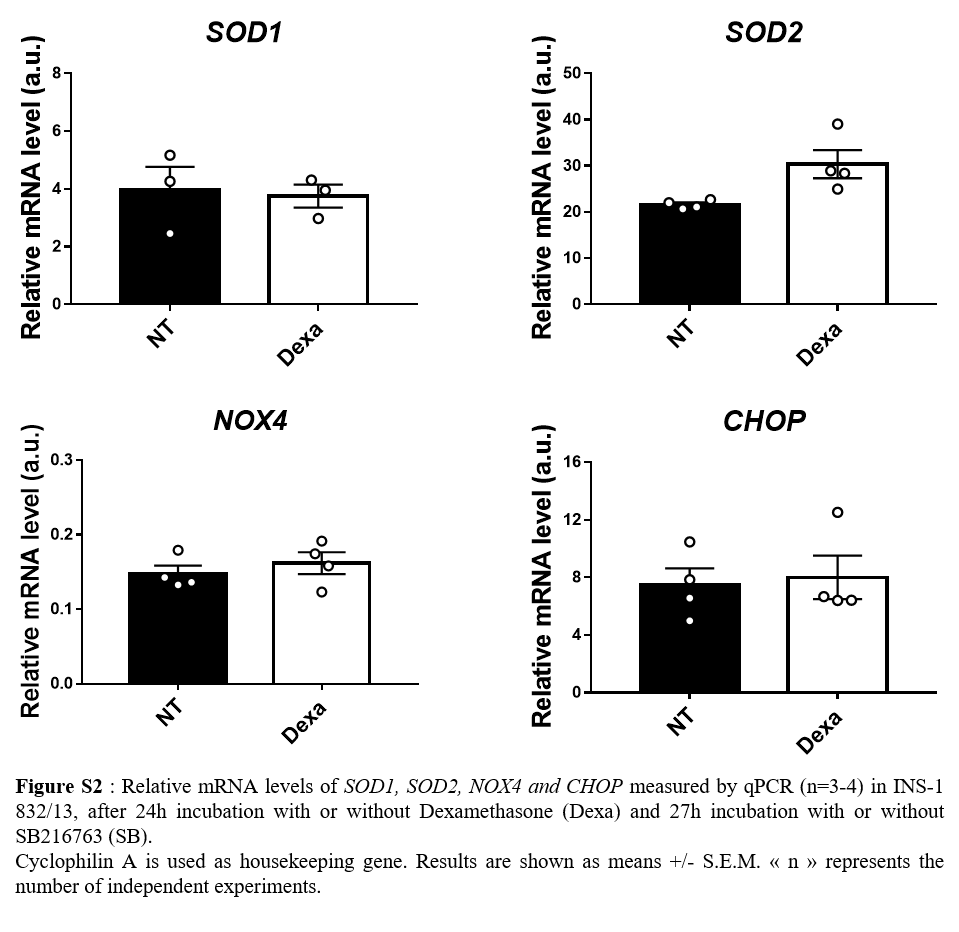

Supplement: Supplementary file 2 — Supplemental Figure 2 [file 41419_2021_4419_MOESM2_ESM.tif]
